# Genetic similarity of biological samples to counter bio-hacking of DNA-sequencing functionality

**DOI:** 10.1038/s41598-019-44995-6

**Published:** 2019-06-18

**Authors:** Mohd Siblee Islam, Stepan Ivanov, Eric Robson, Tríona Dooley-Cullinane, Lee Coffey, Kevin Doolin, Sasitharan Balasubramaniam

**Affiliations:** 1 McAfee Ireland Ltd., Building 2000, City Gate, Mahon, Cork, Ireland; 2 Telecommunications Software and Systems Group, Waterford Institute of Technology, Waterford, Ireland; 3 Pharmaceutical and Molecular Biotechnology Research Centre, Waterford Institute of Technology, Waterford, Ireland; 4 Faculty of Information and Communication Sciences, Tampere University, Tampere, Finland

**Keywords:** DNA sequencing, Computer science

## Abstract

We present the work towards strengthening the security of DNA-sequencing functionality of future bioinformatics systems against bio-computing attacks. Recent research has shown how using common tools, a perpetrator can synthesize biological material, which upon DNA-analysis opens a cyber-backdoor for the perpetrator to hijack control of a computational resource from the DNA-sequencing pipeline. As DNA analysis finds its way into practical everyday applications, the threat of bio-hacking increases. Our wetlab experiments establish that malicious DNA can be synthesized and inserted into *E*. *coli*, a common contaminant. Based on that, we propose a new attack, where a hacker to reach the target hides the DNA with malicious code on common surfaces (e.g., lab coat, bench, rubber glove). We demonstrated that the threat of bio-hacking can be mitigated using dedicated input control techniques similar to those used to counter conventional injection attacks. This article proposes to use genetic similarity of biological samples to identify material that has been generated for bio-hacking. We considered freely available genetic data from 506 mammary, lymphocyte and erythrocyte samples that have a bio-hacking code inserted. During the evaluation we were able to detect up to 95% of malicious DNAs confirming suitability of our method.

## Introduction

In recent years, the field of bioinformatics has undergone a noticeable transformation due to the advancements in both genomics and DNA-sequencing equipment. On one hand, current knowledge of DNA structures contributes immensely to a variety of biological and medical applications from disease screening^1^ to plant^2^ and animal^3^ breeding, to forensics^4^. On the other hand, radical new approaches such as DNA-based data storage^5^ have extended the use of DNA-related technologies. The arrival of the MinION sequencer^6^ and its associated technologies has also significantly increased the accessibility of DNA-analysis to the general public, and in the future will become an essential hardware for a range of industrial applications.

A recent study^7^ has demonstrated a new form of vulnerability that DNA-sequencing can be susceptible to. The study shows how an adversary can insert a malicious payload from a computer script into a DNA sequence of a biological sample. The inserted payload takes advantage of a specific binary vulnerability of software used in the DNA-sequencing pipeline. The pipeline assembles the DNA-structure of a sample from the output of a DNA-sequencing instrument (i.e. FASTQ files). Then, the payload creates and opens a reverse shell to a remote address and port for the adversary to seize control of computational resources hosting the affected software. Though hosted separately from the sequencing instrument, the pipeline is an essential part of the DNA-sequencing process. Control of the pipeline will allow the attacker to eavesdrop on and even sabotage future DNA analyses. This may lead to consequences including misdiagnosis of illnesses, use of wrong DNAs for criminal forensics investigations, or suboptimal animal and plant breeding. In this paper we consider (i) a new scenario of attack on DNA sequencing pipeline, and (ii) input-control for detecting the DNA with encoded malicious code that is used for hacking. The following sub-sections will provide a brief introduction to each of these contributions.

## Physical Transport of Malicious DNA

**Figure 1:**
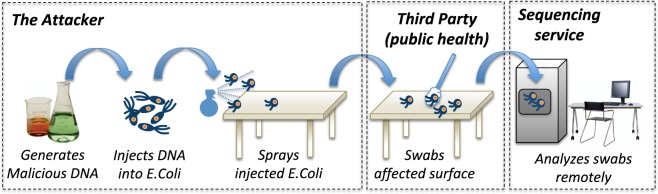
Synthesis of DNA with encoded malicious code, physical transport of the malicious DNA to the targeted remote sequencer.

In this article we consider an end-to-end execution of the attack on the DNA-sequencing pipeline, starting from generation of malicious DNA, to its delivery to the intended target and finally sequencing. The delivery takes the form of spraying the DNA containing malicious code onto different materials, such as a lab coat, glove, or a lab bench. Figure 1 presents an example of our attack scenario where the malicious DNA is injected into *E*. *Coli plasmids*, which in their raw form or injected into *E*. *Coli* bacteria are sprayed on a common surface (e.g. in a restaurant kitchen). The surface is swabbed by a third-party (e.g. during a routine health-and-safety inspection) and the swabs are sent for analysis to an external DNA-sequencing service (e.g. to detect the exact *E*. *Coli* strains present). The DNA-sequencing service is the intended target for the attack. Such scenarios will become more and more prevalent in the future. Due to advances in Cyber-Security, it will become increasingly difficult to gain control over a remote service using software-only vulnerabilities. Therefore, hackers will resort to more sophisticated approaches, such as the attacks we consider in this paper, where malicious code is delivered via DNA samples. These attacks represent a biological version of the *injection* practices used by hackers today.

## Input-Control for Detecting Bio-Hacking

Computer Science has been dealing with injection attacks for some time. Thus, Ron *et al*.^8^ present an overview of injection attacks on NoSQL data-storage systems. Similarly, Tsoutsous and Maniatakos^9^ review the attacks on Embedded Systems. To neutralize the threat identified in^7^, computer science offers a number of solutions. As demonstrated in^10^, certain hardware functionality (e.g. Intel Memory Protection Expansion) may be successfully used to address some of the underlying memory-access issues. However, such solutions are naturally hardware-specific and, therefore, cannot be applied across-the-board. Alternatively, memory-access can be tightened at the Operating System’s level^11^. While this loosens hardware-dependency of such solutions, their applicability is still limited. Finally, at the application level, injection attacks are successfully countered by the input-control techniques. For example^12^, uses input-control as a countermeasure to an injection-based attack on a system managing an electrical grid^13^. Runtime Application Self-Protection described in^8^ is an input-control technique proposed for NoSQL systems. Though built for a particular application, these techniques are compatible with multiple configurations of hardware, middleware, and operating systems. This property is particularly important for protecting a DNA-sequencing pipeline that may consist of a number of diverse computational resources.

**Figure 2:**
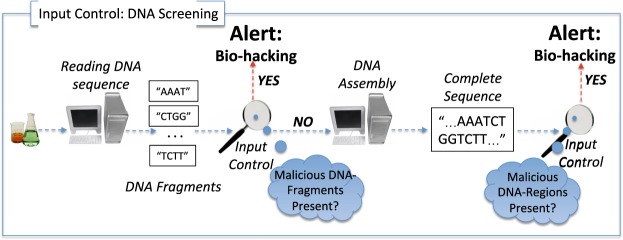
Input control to detect DNA-injections.

In this paper, we evaluate the suitability of using input-control techniques against malicious DNA-injections (Fig. 2). As the main purpose of this article is to establish suitability of input-control, the evaluation is done only for a limited variety of samples. While this is sufficient for the problem at hand, the techniques can be readily extended to account for other samples (see the last paragraph in *Section 2*.*2*). We propose an input-control technique that tightly aligns with the typical stages of the DNA-sequencing process, namely *Reading DNA-Fragments* and *DNA-assembly*^14^. During *the first stage* a complete DNA sample is divided into multiple fragments that are sequenced individually to speed up the overall process. As the result of this step, the chemical structure of each fragment is represented as a string of the “A”, “C”, “T”, “G” letters representing DNA’s corresponding nucleic acids. During *the second stage* the fragments are assembled into the complete DNA-sequence that describes the structure of the entire DNA sample. The two stages present two distinct points where an injection of malicious DNA-content can be detected. The detection algorithm will stop any further processing of the malicious sample, and this is one of the contributions of this paper.

## Methodology

Methodologically, the study presented in this article can be separated into two stages. During the first stage we demonstrate the reality of the threat of bio-hacking. We conducted a series of wetlab experiments to prove that *E*. *coli* bacteria can be successfully used as a viable carrier for malicious code that is encoded into its DNA plasmid. *E*. *coli* bacteria can be used to host, multiply and preserve the code within the environment and/or transport the code to its intended target as part of the physical transport. During the second stage we showed that insertion of malicious code into a DNA sequence reduces its genetic similarity with other organisms from the same biological specie. We used this fact to propose a screening algorithm to detect malicious code in DNA.

### Biohacking as a valid threat

We conducted a series of wetlab experiments to prove the ability of *E*. *coli* bacteria to serve as malicious code carriers. Specifically, we considered the stability of *E*. *Coli* plasmid DNAs. It has been shown that certain *E*. *Coli* plasmids can be used as the media for long-term data storage (e.g. up to 20 days in Accelerated Aging Conditions with temperature of 65C^15^). However, *E*. *Coli* plasmids with encoded malicious code do not portray the typical characteristics seen in bacterial hosts nor has their stability been confirmed over multiple repetition studies. Furthermore, even minor sequencing errors (e.g. incorrect base-calling) have the potential to alter the malicious-code and render it non-functional (e.g. introduce spelling mistakes into the shell code). These errors may occur due to reasons stretching from exposure to UV, heat, chemicals^16^ to phasing effect and other sequencing problems^17^. Therefore, the stability of malicious payload recovery from the manipulated *E*. *Coli* plasmids requires additional confirmation. To do so we reproduced possible steps of a hacker trying to attack a DNA-sequencing service. We first produced *E*. *Coli* plasmids with malicious code integrated into their DNA. Next we evaluated the recovery of malicious DNA from various services sprayed by the hacker as part of the physical transport mechanism.

**Figure 3:**
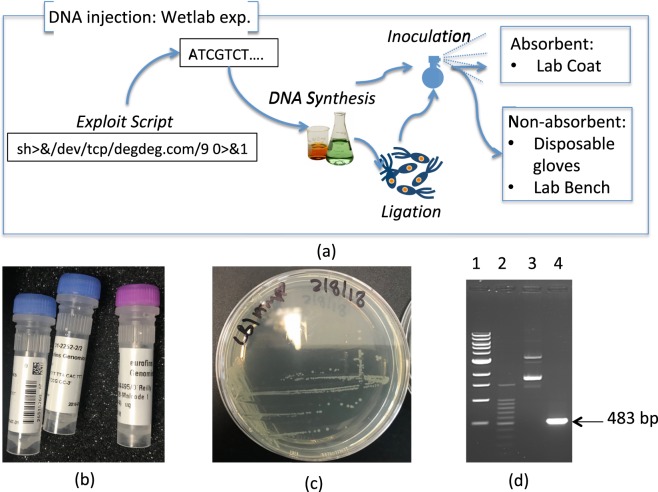
Wetlab Experimental Setup. Gel-Lane content on subfigure (c) Lanes: 1 - NEB 1Kb DNA-ladder; 2 - Promega 100 bp DNA-ladder; 3 - pMal1, complete plasmid; 4 - malicious segment isolated.

Figure 3(a) shows a high-level overview of the experimental design. We successfully inserted via ligation the code from^7^ as a DNA sequence into the plasmid (pEX-A128, see Fig. 3(b)) and designated the final recombinant plasmid as pMal1. This DNA sequence was synthesised by Eurofins Genomics, Germany. The plasmid DNA material was then successfully transformed into a population of *E*. *coli* cells (Novablue strain (Novagen)) as shown on Fig. 3(c). Plasmid DNA (prepared using the Monarch Plasmid MiniPrep kit (New England Biolabs)) and recombinant *E*. *coli* containing the plasmids were separately inoculated onto three surfaces: wooden lab bench, nitrile disposable glove (both non-absorbent) and cloth/labcoat (absorbent). The study aimed to establish if the malicious DNA material could be recovered through swabbing from surfaces several hours past spraying if a hacker was to transport the DNA. In doing so, we model physical malicious code delivery by either live *E*. *coli* or residual plasmids of non-viable bacteria that remained on various surfaces. Controls were carried out to ensure integrity of the wet lab experiments. Negative controls consisted of sterile ultrapure water being added to identical surfaces. That was done to ensure that only DNA material introduced during the experiments would be detectable by our methods. Standard *E*. *Coli* plasmid DNA samples were used as positive controls to confirm credibility of the DNA recovery through swabbing. During controls and core experiments of the study, samples were left on the surface for 24 hours and dried completely before swabbing.

To each surface, 500 ng of plasmid DNA or 1 µl of cell suspension @ O.D._600_ = 12.5 (approximately ten million cells calculated using Agilent Genomics BioCalculator) were inoculated to a 1 cm^3^ area. Both dry and wet swabs (swabs pre-moistened with sterile TE buffer) were used to recover plasmid DNA and *E*. *coli* bacteria from each surface using the cross hatch swabbing technique. The swabs were resuspended in TE buffer and all DNA preparations were quantified using the Qubit3™ Fluorometer and Qubit™ dsDNA BR kit (Thermo Scientific), with cell suspensions measured using a NanoDrop ND-1000™ at an optical density of 600 nm. The isolated material was also subjected to PCR to detect the presence of the DNA material injected with malicious code i.e. pMal1. Oligonucleotides (pEX-For and pEX-Rev) were supplied by Eurofins, MWG operon, Germany. PCR conditions used to amplify Malcode 1 were as follows; each 15 μL PCR reaction mixture contained 7.5 μL Q5® High-Fidelity DNA Polymerase Master Mix (NEB), 15 pmol of each primer and 15 ng pMal1 plasmid DNA. PCR conditions include: 1 cycle of 95 °C for 5 min, 30 cycles of 95 °C for 1 min, 66 °C for 1 min, 72 °C for 30 s, 1 final extension stage of 8 minutes. PCR amplification limits were tested by adding decreasing amounts of pMal1 plasmid DNA to reactions via serial dilution of template. PCR products were analyzed by agarose gel electrophoresis. Figure 2(d) presents complete pMal1 plasmid DNA (lane 3) and PCR amplification of a segment from pMal1 DNA containing the malicious code sequence (lane 4). Sequencing of plasmid insert was carried out in triplicate using the vector-specific primers pEX-For and pEX-Rev, with all sequencing carried out by GATC Biotech, Germany.

### Genetic similarity as a counter-measure

During the genetic similarity analysis we made extensive use of Genetic Signal Processing (GSP) techniques. For an organism (e.g. *E*. *coli* bacterium), GSP works with the string representation of the DNA structure. The structure is presented as a sequence of “A”, “C”, “T” and “G” symbols corresponding to the 4 DNA nucleic acids. The sequence is transformed into a continuous signal (often referred to as *Genetic Signal*) that is then analysed using various Signal Processing techniques. This research used the Voss transformation^18^ to obtain Genetic Signals from DNA strings. The transformation had previously proven efficient in multiple studies on similarities of DNA within biological types, classes and families^19^. Results of the Voss transformation were subjected to Discrete Fourier Transform (DFT). Results of DFT formed the features (specifically Energy Values of DFT’s Frequency Spectrums obtained for DNA Voss transform as presented in the Appendices) that we used to establish dissimilarity between the original and injected DNAs (proven technique, same as in^20^). See Appendix A of the Supplementary Material. The overall transformation represents an arbitrary DNA sequence (may be rather large in length) as a tuple of 20 floating-point numbers. This representation significantly reduces complexity of distance-wise comparisons between DNA sequences. Consider a distance between two DNA sequences whose lengths are m and n. Then, complexity of traditional methods such as Needleman-Wunsch (part of popular BLAST framework) estimated as ***O***(***nm***), while complexity of calculating Euclidean distance in **R**^**20**^ (used in this article) is only ***O***(***1***). This reduction is particularly important when such comparisons may consider multitude of species and their variations.

#### DNA Robustness to malicious injections

Similarity of Voss genetic signals was used to evaluate the effect of malicious code injection of various DNA structures. Thus, initially, the robustness of DNA from mammary, erythrocyte and lymphocyte cells of humans was considered. The three DNA-types are significantly larger in comparison to that of *E*. *coli* plasmids and, therefore, have a larger potential to camouflage the injected malicious code DNA sequences. Therefore, the ratio between the original and malicious DNA codes is lower for human cells, making it harder to identify the injected DNA sequences. This is particularly relevant as our analysis heavily relied on Fourier Spectral Energy values, which tend to overlook smaller fluctuations in signals. Subsequently, results of those analyses were confirmed for *E*. *coli* plasmids.

When investigating robustness of DNA to code injections, we first obtain string representations of real DNAs for each type that we analyze: DNA of *E*. *coli* plasmids as well as mammary, erythrocyte and lymphocyte DNAs of humans. Thus, for the human cells we used 254 mammary, 104 lymphocyte and 48 erythrocyte DNAs obtained from individuals, stored and made publicly available by NCBI database (see Appendix B of the Supplementary Material). For each of the cell-types the DNAs included equal numbers of cancerous mutations and their cancer-free counterparts. For each cell types its DNA strings were modelled with injection of malicious code, where sub-sequence of the original DNA were substituted with malicious DNA sub-sequences similar to that proposed in^7^. Each malicious sub-sequence was generated for a particular shell-command designed to hijack control of the DNA-sequencer. Full list of the commands can be found in the Supplementary Material, Appendix C of the article. Each command was first represented by the binary code of its characters. From the binary code the malicious DNA sub-sequence was then derived by using a simple coding technique, where ‘00’ is substituted with ‘A’, ‘01’ with ‘C’, ‘10’ with ‘T’, and ‘11’ with ‘G’. The ACTG encodings were inserted into the existing DNAs. In each of the DNA a partition of the same size as the encoding is randomly selected, the partition is substituted with the encoding. All of the original DNA and their injected counterparts were amalgamated into a single DNA-pool.

To establish robustness in identifying malicious code injections of DNAs, Case Based Reasoning (CBR), a renowned Data Mining technique was applied. CBR is a technique that mimics the decision-making process of humans such that when an individual needs to make a decision they will first intuitively try to call upon their previous similar experiences^21^, which is continually extended depending on the results caused by each decision. In the exact same way, CBR maintains and updates a collection of previous cases that have been put to it. For genetic similarity assessment, we considered two DNA classes: *Original* and *Injected*. For each class and each DNA-type, we randomly selected a subset of original and injected DNAs from the DNA-pool. The subset would serve as CBR’s previous experience. Euclidean closeness to one of the previous experience cases was used as the basis for classification. Performance of the CBR classification was evaluated for the available DNAs other than those used as previous experience. While this study is only concerned with suitability of input-control techniques, successful use of any classifier is sufficient. However, some of the specific features of CBR are particularly attractive in the context of classification of an arbitrary DNA. CBR does not have an explicit training stage but solely relies on its previously known cases. Therefore, introduction of additional species will only require the cases to be appended with the species’ representative samples. For detailed description of CBR and its use in this study see Appendix E.

## Results and Discussion

Following the methodological stages of this research, the two subsections below present results that we obtained while validating *Biohacking as a Valid Threat* and using *Genetic Similarity as a Counter-Measure*.

### Biohacking as a valid threat

To prove that *E*. *coli* may be used during physical transport of malicious DNA, 500 ng of *E*. *coli* plasmid DNA and approximately 10 million *E*. *coli* bacteria were inoculated to the three surfaces under investigation (i.e. lab bench, glove and lab coat), as described in the previous section. When using wet-swabs to recover dried plasmid DNA from non-absorbent surfaces, 97% of inoculated material was recoverable (see Fig. 3). Using dry swabs, 26% and 16% of dried plasmid DNA was recovered from the bench and glove sites, respectively. When using wet-swabs to recover dried *E*. *coli* bacteria from on-absorbent surfaces ~65% of cells were recoverable. Using dry swabs, 9% of the dried *E*. *coli* population was recovered from the lab bench and glove sites. DNA sequencing of all of the recovered material allowed correct reconstruction of the manipulated DNA. No errors were encountered during sequencing. Neither *E*. *coli* plasmid DNAs nor *E*. *coli* bacteria were recoverable from the absorbent surfaces (i.e. labcoat) to quantifiable levels, regardless of dry or wet swabbing. The detection limits using standard quantification for plasmid DNA and bacteria cells were 1 ng/µl or O.D._600_ = 0.001 respectively.

**Figure 4:**
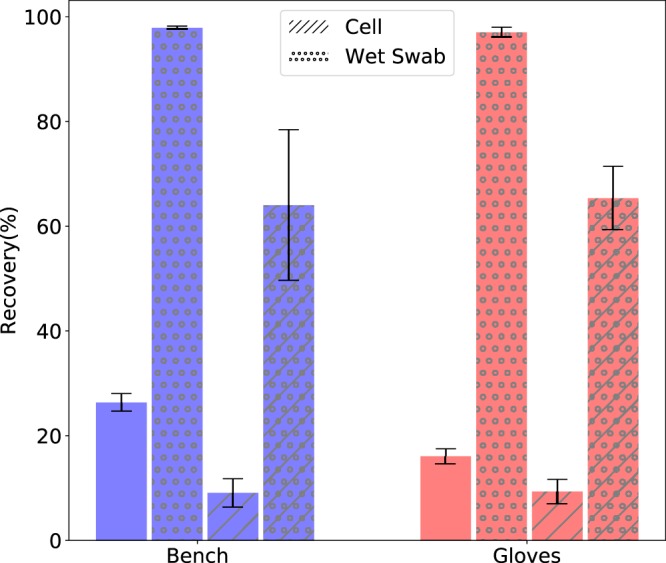
Recovery Rates for Malicious DNA material from non-absorbent surfaces using wet and dry swabbing.

While the results in Fig. 4 paint a picture of relative safety offered by absorbent surfaces, lowering the PCR detection level of the swabbing results did yield some DNA material from the injected *E*. *coli* plasmids and bacteria. Even though, in this case the level of contamination was much lower than what’s required for standard quantification, the amount of manipulated DNA allowed for error-free sequencing and was sufficient to contaminate and compromise a DNA sequencer. To prove that we used a series of *E*. *coli* plasmid dilutions, which were subjected to PCR amplification, as little as 0.1 pg of plasmid DNA as template per 15 µl PCR reaction yielded amplification. As the plasmid used in this study (pEX-A128) is 2,450 bp in size and contains a malicious insert (297 bp), it can be calculated that 0.1 pg of plasmid DNA contains 3.5 × 10^4^ plasmid molecules. Using a conservative estimate of 200 plasmid molecules per cell, only 1,750 cells from the 10 million inoculated on surfaces are needed to yield amplifiable amounts of the malicious DNA. Therefore, it can be concluded that both *E*. *coli* plasmid DNA and *E*. *coli* bacteria possess sufficient capabilities to contaminate and compromise DNA sequencing equipment regardless of the surface type or swabbing method. The persistence, and therefore, stability of cells as a malicious DNA carrier/source of infection would be further augmented if spore-forming cells were used^22^.

### Genetic similarity as a counter-measure

**Figure 5:**
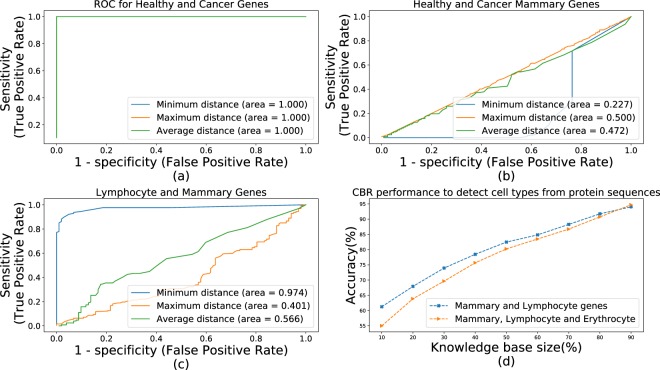
DNA similarity, extended study ROC curve for threshold-classification between (**a**) Healthy/Cancer samples from^20^, (**b**) Healthy/Cancerous mammary and (**c**) lymphocyte/mammary samples from NCBI; (**d**) CBR cell-type classification.

Results of previous studies have shown the use of Fourier Spectrum Energy representation of Voss Transformation for DNA classification. Thus^20^, reports on a particular arrangement for DNA sequences of Healthy and Cancerous samples of human mammary tissue (see *Appendix D* Fig. 9), where all Healthy DNAs belong to a single tight cluster that all Cancerous DNAs lay strictly outside of. However, as results of^20^ are based on a small selection of DNAs, conclusions of^20^ require further validation. Figure 5(a–c) evaluate (via ROC-analysis) threshold-based separation between Healthy and Cancerous DNA using Average, Minimal or Maximal distance to the remainder of Cancerous/Healthy samples. The arrangement reported in^20^ results in exceptional ROC curves with AUC equal 1 (Fig. 5(a)). This does not quite hold for a larger set of DNAs obtained from the NCBI database (Fig. 5(b,c)). Both figures noticeably differ from the results of^20^ in cases of Average and Maximal distances. Both distances express a fairly low predictive capacity (AUC close to 0.5 due to chance), which contradicts one cluster arrangement of Healthy mammary DNAs. However, good predictive capacity of the Minimal Distance (i.e. AUC close to 1 or 0) is an indication of some cluster-like structure amongst the DNAs. This was further confirmed by sufficiently high CBR-classification accuracy obtained for the NCBI’s DNA samples (Fig. 5(d)).

**Figure 6:**
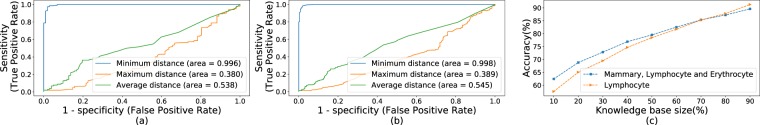
DNA similarity, injection of malicious code from^7^: ROC curves for threshold-classification of injected DNA for (**a**) lymphocyte and (**b**) mammary, lymphocyte and erythrocyte cells; (**c**) CBR classification.

The structure re-established by Fig. 5 further re-affirms the validity of CBR for identification of DNAs with malicious code injections. As cancerous mutations (which typically affect very small DNA-partitions) are sufficient to distinguish Cancerous from Healthy DNAs, much larger code injections should be detectable using the same methods. To confirm that, we first conducted a series of experiments trying to determine if the original malicious code proposed in^7^ could be detected via CBR. Figure 6 presents results for the malicious code injections in mammary, lymphocyte and erythrocyte DNAs. Similar to other figures, Fig. 6(a,b) evaluates the predictive capacity of the three distances. This evaluation closely aligns with the conclusions from Fig. 5. Subsequently, Fig. 6(c) shows results of the CBR classification showing the levels of correct detection increasing up to approximately 90%. While this detection rate may seem low, it is anticipated that higher detection rate will be achieved in a practical setting. The presented results were obtained for various sizes of previously known genetic material exploited by the CBR. To protect a real DNA sequencer, the most complete CBR knowledge (close to 100%) will be used. In our experiments up to 90%-complete CBR knowledge was used, as a portion of known DNAs were required to evaluate the detection itself.

Figures 5(d) and 6(c) provide additional insight in relation to the impact of the set of biological species within which the malicious DNA is detected. The CBR-classification is better for the lesser specie-sets (i.e. *“Mammary and Lymphocyte”* set in Fig. 5(d) and *“Lymphocyte”* set in Fig. 6(c)) when compared to their extended versions (i.e. *“Mammary*, *Lymphocyte and Erythrocyte”* set on both figures). Due to the increased DNA-variability of the extended sets CBR knowledge of a particular size is likely to capture more of the DNA-variability of a lesser specie-set. At the same time, for any specie-set, larger CBR knowledge captures more of the DNA-variability and thus shows better detection accuracy. This can be also seen in Figs 5(d) and 6(c) where all of the detection accuracies increase with increase of knowledge size. However, as the accuracy-growth is naturally bounded, the difference in detection accuracy between lesser and extended specie-sets diminishes with the increase of CBR knowledge size. This will impact on the practical use of the proposed CBR-based detection of malicious DNAs. To ensure adequate detection-accuracy, applications dealing with heterogeneous DNA-sources will require larger CBR knowledge compared to their specialized counter-parts (e.g. Human Lymphocyte-only samples).

**Figure 7:**
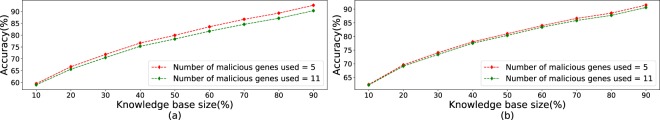
CBR-based detection of diverse malicious DNA in (**a**) lymphocyte and (**b**) lymphocyte, mammary and erythrocyte cells.

Figure 7 summarizes the impact of variability in the malicious code on the detection accuracy. 5 to 11 (see Supplementary Materials) different malicious code samples were injected into DNAs of lymphocyte, erythrocyte and mammary cells. The shape and values on Fig. 7 are noticeably close to those on Fig. 6, which leads us to believe that code variability had little or no impact on the CBR performance. This supports the hypothesis originated from the analysis of Fig. 5. Malicious code exceeds cancerous mutations in size and should be detectable by CBR.

**Figure 8:**
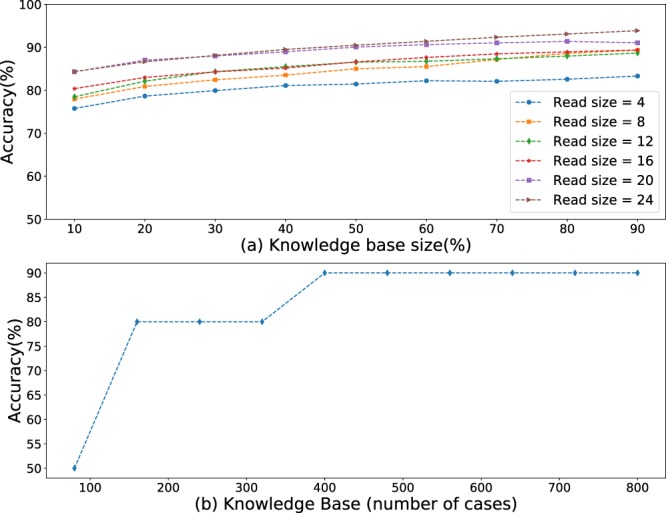
CBR-based detection of malicious content: (**a**) in DNA fragments of human mammary, erythrocyte and lymphocyte DNAs; (**b**) in *E. Coli* Plasmid DNA.

Figure 8(a) investigates the use of CBR for injection classification based on DNA fragments that would be typically formed at the end of the first stage of DNA sequencing. Figure 8(a) looks at how accurate a single DNA-fragment can be identified as coming from a genuine or injected DNA. Note that depending on the DNA sequencer and associated chemistry (e.g., Sanger versus Illumina sequencing or amplicon versus genome sequencing), its user can specify or predict the size of DNA fragments (also called reads, amplicons or fragments to be sequenced) that the original DNA will be assembled into the complete sequence^23^. From the information-security viewpoint, detecting malicious code injections from fragments presents a stronger counter-measure for DNA hacking. Detecting a malicious injection from fragments will preclude any further analysis of the fragments, guarding DNA assembly code from its own binary vulnerabilities. However, the quality of such detection will depend on the size of the DNA fragments. On one hand, for smaller fragments the length ratio between the original and injected components is expected to be higher (compared to larger fragments or full DNA), potentially simplifying the detection. On the other hand, from the perspective of combinatorics, we know that the numbers of all possible “ACTG” string-representations is fewer for shorter fragments. This complicates the detection. This is confirmed in Fig. 8(a), where general classification accuracy increases as the fragment length (or read size) increases. Yet the increase is bounded. Even for fragments of 4 bp, the detection accuracy is higher than that of a full DNA (Figs 6–8). Therefore, the optimal read size is limited, but should not be below 24 bp.

Finally, we investigated the use of CBR to detect malicious code injections into the DNA plasmid material of *E*. *coli* bacteria. Figure 8(b) shows the growth of the detection accuracy with the increase of the knowledge size. As it can be seen from the figure, the growth is more rapid for *E*. *coli* rather than humans. This can be explained by the lower complexity of *E*. *coli* DNA, leading to a lesser representation required to describe the underlying structure of genuine and injected DNAs.

## Conclusions

In this article we have investigated the threat of bio-hacking for modern DNA-sequencers. We have shown that simple organisms such as *E*. *coli* bacteria can be used to transport malicious DNA code to its destination. To protect against this threat, we have proposed a counter measure. We have shown that it is possible to use the existing DNA-similarity of biological species to identify the presence of malicious code within DNA of a particular sample. With the example of lymphocyte, erythrocyte and mammary DNA of humans, we have shown how Voss Transformation and CBR can be used for that identification. The accuracy of the identification increases as more DNA structure information becomes available to the CBR model. Code injections appear to be substantially different to natural mutations of DNA, and variability of the malicious code has lower impact on its identification accuracy. It is beneficial to use DNA fragments to increase the accuracy of identification, where there is an optimal fragment-length to be used. We have also demonstrated a new form of transport for the DNA with encoded malicious code, and that is through various types of materials (e.g., lab coat, glove, or lab bench). This demonstrates that hackers in the future can transport the DNA with the encoded malicious code and swab the samples once they get close to the DNA sequencer, or spread the samples in an environment that will be potentially be swabbed. Experiments have shown that recovery was achievable for materials such as glove and lab bench, but was not very good for cloth.

## Data Availability

All data used during the study presented in the manuscript is freely available in the public domain. The manuscript’s Methodology (e.g. Section 2) outlines the origin of the data. Furthermore, for the convenience of the reader we provide supplementary material that (Appendixes B and C) lists the DNAs and injection codes used.

## Supplementary information

Genetic similarity of biological samples to counter bio-hacking of DNA-sequencing functionality
